# Reaching Low-Income Mothers to Improve Family Fruit and Vegetable Intake: Food Hero Social Marketing Campaign—Research Steps, Development and Testing

**DOI:** 10.3390/nu8090562

**Published:** 2016-09-13

**Authors:** Lauren N. Tobey, Harold F. Koenig, Nicole A. Brown, Melinda M. Manore

**Affiliations:** 1 Extension Family and Community Health, College of Public Health and Human Sciences, Oregon State University, 106 Ballard Hall, Corvallis, OR 97331, USA; 2 College of Business, Oregon State University, 474 Austin Hall, Corvallis, OR 97331, USA; koenig@bus.oregonstate.edu (H.F.K.); nicole.brown@bus.oregonstate.edu (N.A.B.); 3 School of Biological and Population Health Sciences, College of Public Health and Human Sciences, Oregon State University, 103 Milam Hall, Corvallis, OR 97331, USA; melinda.manore@oregonstate.edu

**Keywords:** low-income women, focus group, survey, nutrition, social media, Supplemental Nutrition Assistance Program (SNAP), audience-centered positive messaging, health behavior messages, canned, frozen

## Abstract

The objective of this study was to create/test a social marketing campaign to increase fruit/vegetable (FV) intake within Oregon Supplemental Nutrition Assistance Program (SNAP) eligible families. Focus groups (*n* = 2) and pre/post campaign phone surveys (*n* = 2082) were conducted in intervention counties (IC) and one control county. Participants were female (86%–100%) with 1–2 children at home. Mean FV intake/without juice was 3.1 servings/day; >50% preferred the Internet for delivery of healthy eating information. Participants reported time/financial burdens, low household FV variety and desirability of frozen/canned FV, and acceptance of positive messages. A Food Hero (FH) campaign was created/delivered daily August–October 2009 to mothers through multiple channels (e.g., grocery stores, online, educators). Results showed that the IC had better FH name recall (12%) and interpretation of intended messages (60%) vs. control (3%, 23%, respectively). Compared to controls, the IC were less likely to report healthy food preparation as time consuming or a FV rich diet expensive, and it was easier to get their family to eat fruit. Results did not vary based on county/household characteristics. The FH campaign increased FH awareness and positive FV beliefs. A longer campaign with FV assessments will increase understanding of the target audience, and allow for campaign refinement.

## 1. Introduction

Between 2000 and 2013 Oregon experienced increases in Supplemental Nutrition Assistance Program (SNAP) participation (246%) and food insecurity (3%–4%) [1,2]. Only 2009 data from Oregon Behavioral Risk Factor Surveillance System (BRFSS) provide obesity rates based on income. For Oregon households with income <$15,000 the obesity rate was 28.6%, and for those with incomes between $15,000–$24,999 the rate was 31.8% [3]. These obesity rates are higher than those reported across all income categories ≥$25,000 (20.6%–25.7%). Currently, the prevalence of obesity among all adult Oregonians is 27.9% [4]. Low fruit and vegetable (FV) consumption may be one factor linked to increases in obesity. National research shows that higher quality diets that include FVs are more prevalent in populations with higher socioeconomic status, while rates of obesity and chronic disease are lower [5,6]. In addition, the 2015 Dietary Guidelines for Americans Advisory Committee Scientific Report states that FVs are the only diet characteristic consistently identified with positive health outcomes in every conclusion of the report [7]. Unfortunately, individual and community level barriers limit access to FV by low-income families. Barriers at the individual level include cost, inadequate time for preparation, poor nutrition knowledge, and limited cooking skills, while community barriers include cost, transportation, quality, variety, changing food environment, and societal norms on food [8]. Thus, low FV intake may be an important determinant of obesity and chronic disease risk.

Consistent with the nationwide trend, Oregonians continue to show low intakes of FVs [9]. Although the Center for Disease Control and Prevention reported that Oregon adults eat more FVs compared to other states, intake levels are still below the recommendations of 4–5 cups and 5–6.5 cups of FVs/day for adult (19–50 years) women and men, respectively [9,10]. Using the most recent BRFSS data (2013) only 21% of Oregon adults reported consuming FVs ≥5 times per day [11]. Finally, 2013 BRFSS data show that healthy weight adults in Oregon consume more FVs compared to adults who are obese, 24% versus 17%, respectively [11,12].

Social marketing (SM) can increase healthful eating behaviors, including FV intake [13,14,15]. By definition, SM is “a process that applies marketing principles and techniques to create, communicate, and deliver value in order to influence target audience behaviors that benefit society (public health, safety, the environment and communities) and the target audience” [16]. Based on research supported by the United Kingdom’s National Social Marketing Center, SM effectiveness increases if eight SM benchmark criteria are followed: behavior, customer orientation, theory, insight, exchange, competition, segmentation and methods mix [13,14,17]. These benchmark criteria focus on the target audience in all project phases, including dividing the target audience into subgroups with common characteristics, called segments, to improve targeting.

Behavioral interventions aimed at promoting FV consumption are often grounded in the social cognitive theory (SCT) [18]. Although not all SM campaigns are theory based, those that are frequently use the SCT [19]. The SCT recognizes that people influence their environments just as their environments influence them, thus, this theory focuses on reciprocal determinism or associations between behavior, personal factors, and environment. Important components of the SCT include self-efficacy or the personal belief in one’s ability to do something, observational learning, and a person’s expectancies about the consequences of an action(s) [20].

To address Oregon’s rising obesity rates and low FV intake, Oregon State University (OSU) Extension Service received funds to improve FV intake within low-income Oregon families using SM as part of Oregon SNAP-Education (SNAP-Ed), the nutrition promotion and obesity-prevention component of SNAP. Materials and messages from out-of-state SNAP-Ed funded SM campaigns were tested (described in Section 3.1), but none were a “good fit” for the Oregon target audience. Thus, in 2009 OSU began developing a new pilot SM campaign that now reaches Oregonians millions of times each year through indirect/direct education, SM, and policy, systems and environmental (PSE) change efforts. The PSE efforts focus on local policy changes, using local food systems and improving school food environments. The campaign goal was to increase FV consumption within the Oregon SNAP-eligible population, which is at risk for poverty, food insecurity, low intake of FVs, chronic disease, and obesity.

To our knowledge there are no published studies outlining the formative development steps, implementation, and testing of a SM campaign aimed at increasing low-income families FV intake by targeting mothers with children in the home. Thus, this article describes the steps used to develop, implement, and test the Food Hero SM campaign with SNAP participants, and presents the findings from this process. One of the first steps was to determine our target audience within SNAP that was ultimately identified as mothers with their children living in their home. The campaign development strategy was to identify how best to empower and support SNAP-eligible Oregonian’s to overcome perceived and actual barriers to FV consumption and reinforce existing positive behaviors. Our campaign development objectives were to (1) define a target audience and develop a robust understanding of them; (2) identify barriers and what “moves and motivates” them regarding FV intake; and (3) use data gathered to create and test a pilot SM campaign (2-months) that could eventually result in positive behavior change. We hypothesized that a multi-channel, targeted and segmented short pilot SM campaign would increase awareness of the campaign and positive beliefs about FV intakes. We did not expect changes in FV behaviors in 2 months, since the goal was to further refine the campaign through formative/process evaluation and then assess changes in FV intake.

## 2. Materials and Methods

### 2.1. Study Overview

**Figure 1:**
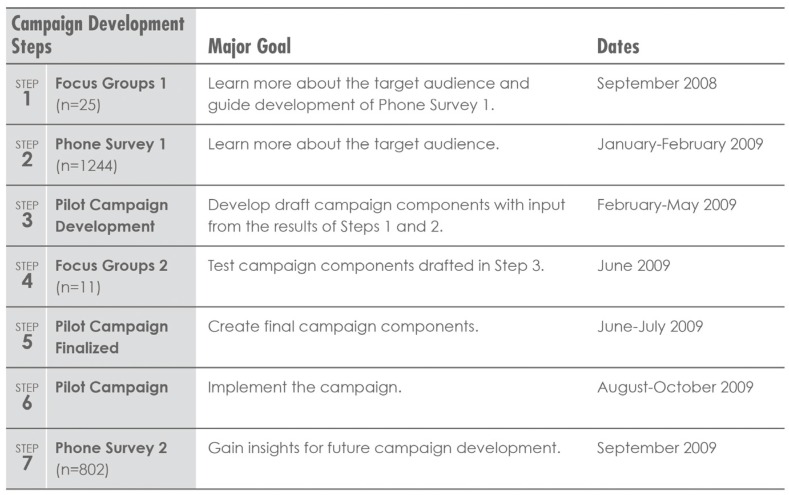
Food Hero development steps, goals and timeline.

Two contractors were hired to add expertise to the campaign development team, a business research firm and a SM firm. The research firm (*Close to the Customer Project*, OSU’s School of Business) assisted with writing research phone surveys (PS) and focus group (FG) questions, managed all phases of participant recruitment, lead the FGs, produced verbatim transcripts of FG discussions and analyzed FG data for common themes, conducted PSs, analyzed the data, and created data summary reports. The SM firm (*EnviroMedia*, Portland, OR, USA) assisted with brand creation, campaign message development, project management, and gave input into PS and FG questions. The campaign development included the eight elements of the SM benchmark criteria [17]. OSU’s nutrition researchers gave input on all steps of the project (Figure 1).

**Figure 2:**
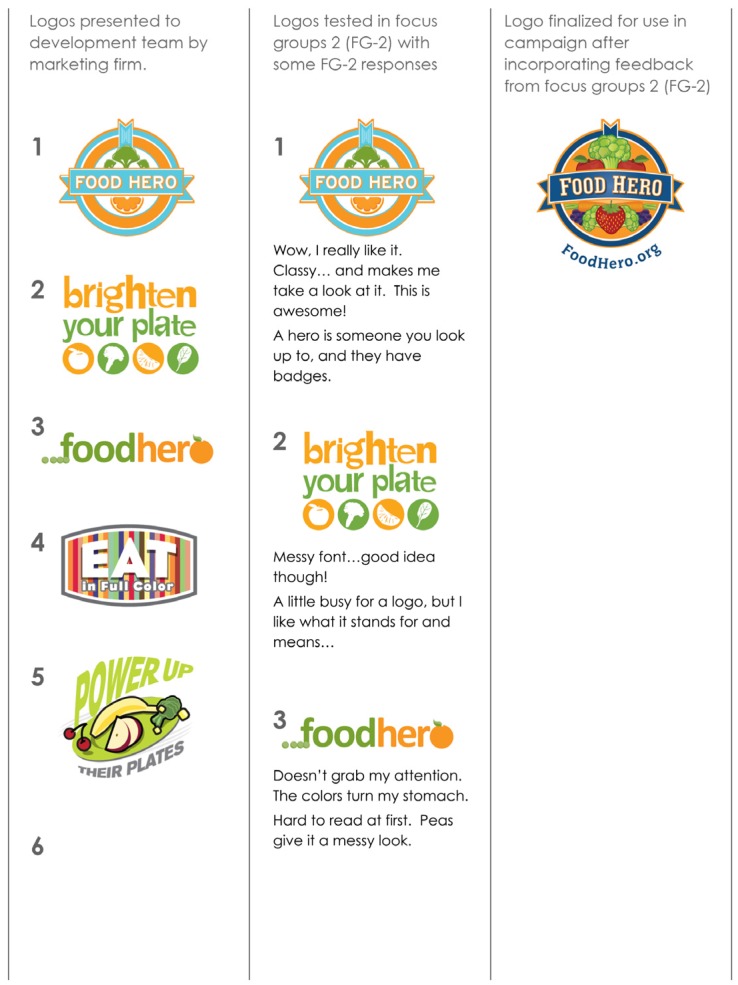
Process for the Design and Testing of Food Hero Logos (Focus Groups 2).

All research was conducted with SNAP participants in select Oregon counties. The timeline and steps of the research and campaign development are outlined in Figure 1. Pre-campaign research included conducting FGs (Step 1: FG-1; *n* = 25 participants), which were used to help design a pre-campaign PS, including FV belief and barrier questions (Step 2: PS-1; *n* = 1244 participants). The PS’s also included the validated BRFSS FV questions to assess FV intake [21]. In addition, the FGs provided information related to health priorities, beliefs and experiences. Using the results from FG-1 and PS-1, Step 3 developed the campaign, including creating draft campaign names, logos, messages and delivery channels (Figure 2 and Table 1). Key components of the SCT aligned with the FG-1 and PS-1 results; thus, the SCT was used to inform campaign development/implementation (Steps 3–6). As second set of FGs (Step 4: FG-2, *n* = 11) were used to test messages and components of the developed campaign. In Step 5, final campaign materials and delivery channels were determined/created and then implemented in Step 6. The campaign was delivered through multiple channels, including a web site, direct mail, billboards, web banner ads, grocery store demonstrations, grocery cart ads, and county SNAP-Ed educators delivering the campaign using Food Hero Community Kits [22]. The community kits were designed to provide locally adaptable campaign tools and materials for OSU’s Extension county educators and their partners. The goal of the community kit was to assure that comprehensive educator/partner Food Hero promotion occurred concurrently with other campaign communication channels. Public relations efforts throughout the campaign included television and radio interviews, a family video makeover contest, and social media postings (i.e., YouTube and Facebook). The Food Hero social media project is described elsewhere [23]. Due to the time left in the project funding cycle, the campaign could only run for 2-months. Near the end of the campaign, a second PS was conducted to test for campaign awareness and FV beliefs, and to gain further insights for future campaign development (Step 7: PS-2; *n* = 802).

### 2.2. Study Design

**Figure 3:**
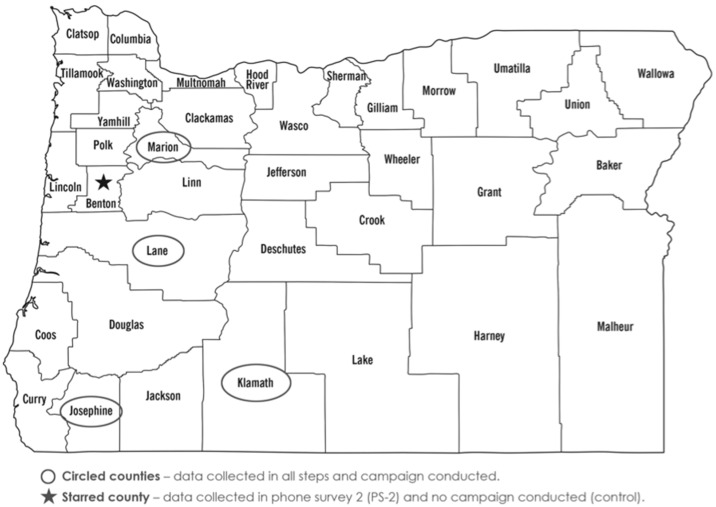
Oregon counties where formative research and pilot campaign were conducted (circled names).

From Oregon’s 36 counties, four (rural = 2; metropolitan = 2) were selected for data collection and to receive the Food Hero campaign (Figure 3). Inclusion criteria for all groups included current SNAP-Ed series of adult/family classes at multiple sites, availability of multiple media buy options, adequate population base for data collection, and SNAP staff available for assistance. Due to cost constraints, only one control county (Benton) was selected for PS-2 data collection because it had qualities of Oregon’s rural and metropolitan counties (i.e., mid-sized population, ethnic diversity, away from a major highway). Benton county residents were also less likely to see Food Hero campaign billboards because it is located off Oregon’s only major highway.

### 2.3. Participants and Recruitment

All FG and PS participants were randomly selected from a list of all English speaking SNAP families living in the targeted counties. The Oregon Department of Human Services (DHS) provided the names and phone numbers, either landlines or cell. For PS-1 it was determined that 250 responses were needed per county, which allowed for counties to be analyzed by themselves with a margin of error ±5% on dichotomous questions (Table 2). Oversampling was done to assure adequate sample size. Calls were made and consenting participants self-verified that they met the following inclusion criteria; SNAP enrollment, county residency, being the primary household food preparer, and having one or more children <18 years living in their home.

Rural versus urban responses from FG-1 and PS-1 were similar, thus, FG-2 was conducted in only one county (Marion) (Figure 3). Participants in FG-1 and PS-1 were 86%–92% female, thus, only females were recruited for FG-2. Due to DHS privacy requirements we do not know if participants overlap between or within the FG and PS samples, but FG participants represented less than 2% of the total study sample. Rural and urban responses on PS-1 were similar; therefore, the sample size for PS-2 was decreased. The research was approved by the Oregon State University Institutional Review Board and participants gave informed consent.

### 2.4. Instruments and Procedures

#### 2.4.1. Focus Groups

For each FG 10-12 participants were recruited via phone calls, then mailed information packets, and received a reminder called prior to the meeting. Compensation was given for the 2-h FG meetings (FG-1: meal +$50; FG-2: meal +$25). *Close to the Customer Project* provided trained facilitators who lead participants through a series of pre-determined questions designed to elicit information regarding FV intake by the family, beliefs regarding FV intake, and barriers to FV intake. Questions for FG-1 focused on campaign development, while questions for FG-2 focused on testing potential campaign messages and components. Follow-up and probing questions were used as necessary, and participants engaged in writing responses and small group discussion.

#### 2.4.2. Phone Surveys

PS-1 data were collected early in the pre-campaign, while PS-2 was conducted the last 2-weeks of the campaign (Figure 1). Both PSs used identical recruitment procedures. Due to DHS privacy requirements, all potential participants received a letter from DHS with the following information: (1) the purpose and time commitment of the survey; (2) that DHS had given their phone number to OSU; and (3) they might receive a phone call asking them to participate in the survey. Trained PS interviewers conducted the surveys, which included moving systematically through a series of questions (PS-1: 70 questions, 24% response rate, interview time ~10 min; PS-2: 32 questions, 11% response rate, interview time was ~5.5 min). If the call resulted in no answer/answering machine, four more call attempts were made before discarding the telephone number. Participants received no compensation. Using mock phone interviews, interviewers practiced and PSs were pre-tested for length, time and clarity. No changes were made to the survey based on the mock interviews. Rural counties were disproportionately oversampled to assure sufficient responses.

### 2.5. Data Analysis

Analysis of FGs was iterative and occurred at several levels by the same four trained researchers who attended all FGs. The research team debriefed immediately following each FG, identifying key insights, themes, and unusual findings. Next the FGs were transcribed and transcripts were read individually by each of the researchers who coded and identified emerging themes. Researchers then met as a group to agree upon and define themes. Once themes were identified, researchers identified quotes and organized them according to thematic content. The focus groups were “exploratory” in nature and the insights gained from the groups informed the construction of the PS questionnaires.

For the PSs, summary statistics were generated for demographic data. ANOVA was used to determine if differences existed between PS-1 and PS-2 and PS-2 and control PS-2. Results showed no differences between surveys based on demographic data (county of residence, single/dual parent household, household size); thus, data from the intervention counties were combined for further analysis. Independent *t*-tests were then used to compare changes in variables/belief statements pre/post campaign using predetermined hypotheses based on campaign expectations.

## 3. Results

Demographic data for FG and PS participants are given in Table 3.

### 3.1. Focus Groups and Phone Survey 1

Step two of the SM benchmark criteria states that a strong understanding of the audience should be developed by combining data from different sources; thus, results from FGs and PS-1 were combined into themes and discussed below. FG and PS-1 results were grouped into five themes/outcomes: (1) audience demographics and characteristics; (2) self-reported FV intake and consumption; (3) grocery shopping and meal preparation habits; (4) preferred communication channels; and (5) responses to previously developed campaigns and messages.

#### Main Themes

Theme 1: Audience demographics and characteristics.

Overall, the participants were female (86%–100%), mean age range 34–43 years, single parents (39%), working outside the home at least half-time (33%–36%) and had 3–4 people in the household (36%–65%), including 1–2 children (64%–76%). PS-1 results showed that single adult households faced additional time and money constraints compared to two adult households. One FG participant said, “Money, I worry about money, I worry about it all the time, it’s a big concern so I try to keep it from my kid so she doesn’t see it.” Working and stay-at-home parents also expressed time constraints.

Theme 2: Self-reported fruit and vegetable intake and consumption.

Based on PS-1, the mean servings/day of vegetables was 1.7, while the mean serving/day for fruit was 3.1 with juice and 1.4 serving/day without juice. Most PS-1 participants (70%) self-reported being knowledgeable about preparing vegetables and found it easy for their families to eat enough vegetables (60%) and fruit (80%). FG results were similar and responses from the FGs are included below:

- “Potatoes are not just for dinner, we can have them for breakfast and lunch.”
- “Carrots are great. I have them as a snack.”
- “They are a good snack.”
- “My kids like the baby carrots so instead of buying the big long bugs bunny carrots they’ll eat the baby carrots.”
- “I always have them [bananas] in my house. They are a year round fruit for the most part. When they get home from school they have one.”

When shopping for groceries, PS-1 participates reported that “low price” was “very important” (67%), while “fast to prepare” (14%) and “easy to prepare” (15%) were less important. Over half (51%) of the PS-1 participants agreed that a diet rich in FVs was expensive. FGs participants often mentioned price as being important when shopping, “I only buy cereal when it is on sale or with a coupon.” Being fast to prepare was also mentioned, but less often, “We eat pasta ‘cause it’s fast.”

Participants from PS-1 and FGs reported that fresh produce was the preferred choice for being most nutritious (i.e., not containing extra sugar/salt or losing nutritional value from canning or freezing), while frozen foods were a good second choice, and canned were less preferred. In PS-1, 39.8% of participants disagreed with the statement “frozen vegetables are just as healthy as fresh,” and 63% disagreed with the statement “canned fruit is just as healthy as fresh”. Some FG participants said canned was “ok” if nothing else was available, while others said they do not buy canned FV because they thought canned FVs had lower quality and less nutritious, especially if they are “cheap” or on sale. The FG participants also perceived fresh as being costly, since fresh FV are perishable and seasonal.

FG participants could name a wide variety of FVs, yet PS-1 participants reported consistently purchasing the same FVs (e.g., apples, bananas, corn, beans and broccoli). Barriers to eating a larger variety of FVs, reported by FG participants, included inexperience in planning and preparing meals, cost and time commitment, seasonality, and coping strategies to get children to eat them. Some responses are included below:

- “I make stir fry because my son’s not a big vegetable eater but he will eat it (stir fry). Now he likes broccoli, caluliflower, stuff like that.”
- “I buy fruits and vegetables in season…but I buy broccoli year round.”

Theme 3: Grocery shopping and meal preparation habits.

Overall, FG participants presented themselves as savvy shoppers, yet found it difficult to provide nutritious meals for their families even with the additional SNAP benefits. Participants indicated they knew how to eat healthfully, wanted their families to have nutritious food, and reported they read product packaging and nutrition labels. One FG participant stated, “I only buy things for her that are good for her. If she wants sweets we make our own and use less sugar.”

When asked about preparing dinner, PS-1 participants (46%) reported they ‘rarely’ had help. Overall, 80% agreed with the statement, “I would like to serve more balanced meals to my family”, while 54% disagreed with the statement “healthy food is time consuming to prepare.” Some FG participants acknowledged they have little experience with, or knowledge of planning and preparing healthy meals. For example one participant said, “I think I wish that one of them things I could do is to make a more balanced meals, I try really hard but I don’t have all the knowledge that I need to make the balanced meals.”

Theme 4: Preferred communication channels.

Half of PS-1 participants indicated that the Internet was the preferred method for finding information on healthful food choices, followed by grocery stores (16%), and magazines (12%). The Internet was the preferred method for participants living in both single adult (51%) and multiple adult households (56%). Those individuals between the ages of 25–34 years reported the Internet as the preferred option (58%) versus those 45–54 years (40%) or 55–64 years (30%). PS-1 participants reported using the following sources for cooking tips and ideas: Internet (~28%) friends and family (~25%), cookbooks (~12%) and television (~12%).

FG-1 and FG-2 participants were asked different questions related to communication channels. When FG-1 participants were asked about US Department of Agriculture (USDA), Food and Nutrition Service materials (e.g., SNAP, The Special Supplemental Nutrition Program for Women, Infants, and Children (WIC), and school materials) as healthful eating resources they reported these resources were not motivating and perceived them as having a “government look”. FG-2 participants were asked about Internet usage and on-line social networking opportunities. They reported wanting healthful eating websites to provide actionable information, including food advice, healthful recipes, and preparation shortcuts. Specific suggestions included “fast and easy recipes that kids will eat”, “recipes with 5 ingredients or less”, “gardening tips (when to plant)”, and “age appropriate sections so their children could look for items they could cook.” Facebook was the participant’s main social media site, more “content” and “more of a network” compared to other sites, and Google their main search site. Finally, the majority of participants stated they like to watch cooking shows (i.e., on the Food Network) to learn about new recipes and preparation tips; however, many felt these recipes were too difficult to replicate. For example, one participant said, “sometimes ingredients are too complicated…”.

Theme 5: Responses to previously developed campaigns and messages.

Materials and messages from out-of-state SNAP-Ed funded SM campaigns were tested in FG-1 to determine if they were a “good fit” for our campaign and if the materials could be used “as is” or in a “modified format”. Participants were asked to review two campaigns: Iowa’s “Pick a Better Snack” and California’s “Champions for Change”. They preferred Iowa’s campaign and indicated that it featured produce items they would buy/eat and responded positively to its message “Ready-to-Serve.” For example, one mom expressed, “If you think about a snack versus a candy bar or bag of chips…get some grapes, you don’t have to unwrap them, its easy, it’s not that hard to eat healthy.”

However, Iowa’s “Pick a Better Snack” message “It’s That Easy” evoked some negative responses indicating it is not always easy to eat healthfully. One comment was, “It’s condescending! It’s not that easy! Fruits and veggies are expensive, so it’s not easy to buy them.”

Reponses to the “Champions for Change”; campaign were less favorable. Participants felt that this campaign placed primary responsibility for family food choices on mothers, and that it featured overbearing mothers, with such messages as “My Kitchen, My Rules”. They also commented that the kitchen is a family space and not solely mom’s space. Participants expected to see media ads for these two campaigns at WIC clinics; however, earlier they indicated that nutrition materials at these clinics did not change their eating behavior. Finally, they reported that both campaigns would have a greater likelihood of changing their FV purchasing behaviors if delivered in a grocery store. Although some FG participants mentioned they would like these messages to come home from school in their children’s backpacks, earlier in the FGs they said these materials were treated as junk mail.

After reviewing FG-1 and PS-1 results, it was determined that a new SM campaign should be designed. Components of a new campaign were created including two mock-up campaign names, three logos, and seven messages (Figure 2, Table 1). FG-2 was used to test these components. Of the name/logo combinations tested, Food Hero name/logo number 1 tested best (Figure 2). Participants saw themselves as being “Food Heroes” and talked about making their children and others “Food Heroes.” They gave feedback on colors and logo use. Participants were asked to rate FV messages (Table 1) on a scale of 1–10 (1 = very bad; 10 = very good). They preferred positive messages that promoted good role modeling within the family, and did not like negative messages or messages that emphasized their financial struggles. They readily accepted messages that were perceived as genuine, and quickly rejected messages they considered disingenuous regardless if they were true or not. The highest rated message was “canned, frozen, or fresh they all start out the same.” The other messages are presented in rank order in Table 1.

### 3.2. Phone Survey 2

The following two key themes/outcomes emerged from PS-2: 1) participant recall and awareness of the Food Hero campaign and 2) beliefs about FVs.

#### Main Themes

Theme 1: Participant recall and awareness of the Food Hero campaign.

Within the intervention counties, 12% of participants recalled the Food Hero name vs. 3% of participants from the control county. When participants were asked the question, “When you hear the phrase “Food Hero” what is the first word or phrase that you think of?”, 60% in the intervention counties correctly interpreted the intended meaning of Food Hero vs. only 23% in the control county. Participants associated Food Hero with eating nutritiously, being a good role model, and eating FVs. For the intervention counties, 68% recalled hearing or seeing at least one of the campaign messages (Table 1). The message with the greatest recall (58%) was “Give them more of the good stuff”. SNAP households in intervention counties received this message via direct mail, and on billboards in all counties except Josephine County due to a lack of SNAP-Ed approved billboard locations.

Theme 2: Beliefs about fruits and vegetables.

Campaign participants were asked to respond to a series of eight belief statements regarding FV use (Table 2). With data combined for all intervention counties, three of these belief statements showed significant change from PS-1 to PS-2 (*p* < 0.05). First, participants reported it was easier to get their family to eat fruit. Second, they were less likely to report that it was time consuming to prepare healthful food. Third, they were less likely to report that it was expensive to eat a diet that included “a lot of” FVs.

When comparing PS-2 for the intervention versus the control county for the same eight belief statements (Table 2) the results were similar except for two belief statements (*p* < 0.05). First, the control county had significantly lower confidence in serving balanced meals for their family. Second, they were less likely to report that canned fruit was just as healthy as fresh fruit.

## 4. Discussion

This study describes the development, implementation and testing of a SM campaign that targets low-income mothers with the goal of increasing child/family FV intake. Three key outcomes resulted from the campaign development: (1) segmentation of the target audience into online and non-online groups, and the inclusion of a secondary target audience (e.g., children); (2) showcasing all forms of FVs (i.e., fresh, frozen, canned, dried and juice); and (3) focusing on positive, actionable messages.

The campaign audience was determined to be SNAP-eligible mothers with children, as they were most often the food buyer/meal preparer in the household. This decision supports data showing that in the US, women do the bulk of these household activities [24,25]. More than 50% of PS-1 participants (86% female) reported wanting to obtain healthful eating information from the Internet, including both single/multi adult households and those age 25–34 years, while the other half did not mention the Internet. Thus, the campaign audience was divided into two segments to address their different communication needs (internet users and non-internet users). The primary segment (53.3%) was mothers who mentioned going online for healthy eating information (i.e., FoodHero.org web site and a campaign social media platform). A secondary segment was for mothers who did not mention going online for healthy eating information (46.7%) (i.e., contents of the community kit such as the printed Food Hero monthly magazine and media buys in grocery stores). It is likely that the segment of mothers who go online for healthy eating information has grown as current Internet usage by US adults with a household income <$30,000/year is 77%; 17% higher than in 2009 [26,27]. Social networking site usage of online adults US adults with a household income <$30,000/year is 79% [28].

The secondary campaign audience was determined to be children of the target mothers who live with their mothers and ≤18 years. In the FGs and PSs mothers talked about engaging and empowering their children with regard to cooking and eating healthy foods. For example, mothers talked about wanting to be healthy eating role models for their children and including children in cooking and increasing children’s acceptance of healthy foods. Similar to our results, Treiman et al. [29] found that Maryland WIC participants taking part in FG interviews (*n* = 239) were highly motivated to be good role models for their children, as were mothers (*n* = 140) who participated in the USDA Maximizing the Message FG project [30]. Campaign components developed for the secondary campaign audience were designed to empower children to be positively involved in healthful food shopping and meal preparation (i.e., inclusion of “kids can” tips in the Food Hero Monthly magazine, a statewide search for children’s artwork to feature in the annual recipe focused calendar, a large segment of Food Hero on the ground cooking/tasting events occurring in schools).

Recipes became the primary product of the campaign since the FG results indicated mothers wanted quick and healthy recipes their children would enjoy. Research shows that as in-home food preparation increases (including within food insecure households) FV intake also increases [31,32]. A major campaign strategy was to include multiple forms of FVs in Food Hero recipes, thus, promoting different types of affordable FVs. This strategy was adopted to lower the consistently reported time/financial burdens and low household variety of FV types used in the home. Incorporating a greater variety of vegetables into the diet has been shown to increase vegetable intake and overall diet quality, including the diets of low-income women [33,34].

FG-2 participants responded well to messages promoting frozen/canned FV. Research shows that frozen/canned FV are cost effective and healthy, and in most cases only take minutes to heat up and serve [35,36,37,38,39]. However, our participants, in alignment with the USDA Maximizing the Message project participants, felt frozen/canned FV were less healthful and desirable than fresh despite the majority of them reporting “low-price” as a shopping priority and 46% reporting “healthy food is time consuming to prepare” [30]. “Low-price” and “time” have been reported by others as food buying influencers and ‘cost’ a barrier to FV consumption; yet, canned FV intake continues to decline in the US [40,41,42,43,44,45]. Canned FV purchases are highest for high-income households, and decline with children in the home [43]. Our results are important since little research exists to describe why mothers prefer fresh FV to canned or frozen. Similar to our results, Black et al., [46] found that Maryland WIC participants (*n* = 223) preferred fresh FV vs. canned due to taste (e.g., disliked the “canned taste”) and did not feel comfortable feeding canned to their children. For a low-income audience, actionable strategies to increase intake of healthy, low cost canned and frozen FV along with fresh FV might increase FV consumption, including variety of FV.

Based on the pre-campaign research, campaign development focused on actionable, positive and empowering messages and materials that were genuine, non-governmental looking, and appeared to be delivered by a trusted friend or family member. Participants clearly indicated they understood the importance of healthful eating and were willing to include more FVs in their diets yet, like most US adults, they were not meeting the current FV recommendations (≥ 5 cups FV/day) [11]. Thus, the SM campaign focused on actionable messages that would help mothers increase FV intake amidst their time/financial limited resources and desire to be healthy role models, versus a focus on the importance of FV. Similarly, the USDA Maximizing the Message project also found that moms responded well to actionable, genuine messages, that would fit into their busy lives [30]. Campaign messages were structured to be applicable to the target population’s situation, aiming to limit/lower their household burdens, and not emphasizing their financial struggles (i.e., those rated higher in Table 1). Our use of positive (gain-framed) vs. negative (loss-framed) messaging is an effective way to promote health behaviors especially for adherence to preventative behaviors like healthful eating [47,48,49,50].

Results showed that participants in the intervention counties recalled awareness of the campaign. When comparing PS-1 to PS-2 questions examining beliefs about FV (Table 2), results were either positive or neutral, providing some feedback on the potential impact of the campaign. We do not know if the Food Hero campaign was responsible for the positive pre/post changes as other factors may have influenced participants’ FV beliefs (e.g., other campaigns, changes in the economy, or seasonality). However, the results are useful for future comparisons and have aided in the refinement of the campaign.

### 4.1. Study Strengths

Overall, this research has four key strengths. First, to our knowledge this is the first SM campaign, aimed at FV intake for SNAP-eligible families by targeting mothers, that has outlined the formative steps used for campaign development, including implementation and testing of a pilot campaign. Research on formative assessment of FV SM campaigns differed from ours in their breath (national campaigns with broad audiences), focus (local campaigns and audiences), audience (focused on low-income parents not mothers), or provided no assessments (focused on mothers but no assessments reported) [15,51,52,53,54,55,56,57]

Second, only SNAP participants were recruited using a random sample provided by Oregon’s DHS. Third, mixed methods were used to gain in-depth understanding of our target mothers. Fourth, rural and urban counties were included to determine if the same campaign worked for these different populations.

### 4.2. Limitations

Study limitations are primarily related to funder guidelines: (1) The project needed to be completed within 1-year, which limited key development factors (e.g., in-depth message testing, cognitive testing of the belief questions, comprehensive website development, pilot campaign length); and (2) Only direct education and SM were allowed with SNAP-Ed funds, thus, at the time of development we could not fully engage in PSE change strategies. Consequently, we could only pilot test the Food Hero SM campaign for 2 months, with plans for later assessments that would measure change in FV behavior. Finally, the outcomes from the Food Hero SM campaign may not be generalizable to other population groups, since we had a specific target group of low-income mothers with children in the home and who spoke English.

### 4.3. Implications for Research and Practice

As a result of this research, the Food Hero SM campaign has evolved and is now a statewide campaign. Currently over 125,000 unique users visit FoodHero.org each month, with over 2.2 million page views in 2015. Since the research described in this paper was conducted, ongoing FGs and PSs with the Food Hero primary and secondary audiences have helped to further refine our campaign components. Behavior change assessments are being done to determine changes in FV intake, PSE strategies incorporated, and both English and Spanish language versions of the campaign are available.

Others working to increase FV intake with low-income mothers can use the insights learned from our target population for their own research/programs. Additionally, we provide an example of potential steps to take to create a new campaign and/or test existing campaigns. Finally, perceptions of different forms of FVs gained from this research can be used to frame FV messaging in health promotion.

## 5. Conclusions

For SM to demonstrate positive behavior change outcomes, campaigns must follow evidence-based practices, such as being designed and tested for a specific audience. Following such processes will allow research to be compared and aggregated. To use an existing SM campaign, it is imperative that the intended audience is a close match to the audience for which the SM campaign was designed.

## Figures and Tables

**Table 1:** Food Hero Messages: Tested and Used in Pilot Campaign.

	Message Priority Given by Participants from Highest (1) to Lowest (7) Preference
Messages Tested in Focus Groups 2 (FG-2)	Canned, frozen, or fresh they all start out the same. Kids would pick candy for every meal, good thing you’re in charge. Canned and frozen fruits and vegetables make your money go further. Buying canned and frozen helps you get more for less. An apple a day is not that far away. Fruits and vegetables are within reach. In these tough economic times get more of a good thing.
Messages Used in Campaign and then Tested in Phone Survey 2 (PS-2)	Give them more of the good stuff (direct mail and billboards) Brighten your plate (website banners, refrigerator magnet grocery store reinforcement).

**Table 2:** Mean responses across all counties to belief statements about fruit and vegetable intake from Phone Survey I (PS-1, *n* = 1244) and Phone Survey 2 (PS-2, *n* = 802) ^1^.

Belief Statement	PS-1 Mean ± SD	PS-2 Mean ± SD	*p* Value (PS-1 vs. PS-2)	PS-2 Control Mean ± SD	*p* Value (PS-2 vs. Control)
1. I know how to prepare many different vegetables.	4.25 ± 1.06	4.25 ± 1.08	*p* = 0.50	4.23 ± 1.16	*p* = 0.44
2. I want to serve more balanced meals to my family.	4.33 ± 1.03	4.35 ± 1.09	*p* = 0.35	4.13 ± 1.14	*p* = 0.03 ^2^
3. Canned fruit is just as healthy as fresh fruit.	2.17 ± 1.17	2.21 ± 1.25	*p* = 0.30	1.93 ± 0.96	*p* = 0.01 ^2^
4. Frozen vegetables are just as healthy as fresh.	2.90 ± 1.37	3.00 ± 1.30	*p* = 0.065	2.91 ± 1.25	*p* = 0.24
5. It is easy to get my family to eat vegetables.	3.73 ± 1.30	3.82 ± 1.26	*p* = 0.055	3.79 ± 1.22	*p* = 0.38
6. It is easy to get my family to eat fruit.	4.37 ± 1.00	4.49 ± 0.90	*p* = 0.005 ^2^	4.43 ± 1.06	*p* = 0.27
7. Eating a diet that includes a lot of fruits and vegetables is expensive.	3.42 ± 1.39	3.17 ± 1.45	*p* = 0.0005 ^2^	3.06 ± 1.30	*p* = 0.21
8. It is time consuming to prepare healthy food.	2.45 ± 1.31	2.29 ± 1.27	*p* = 0.005 ^2^	2.45 ± 1.28	*p* = 0.09

^1^ Responses were recorded on a five point scale where 1 = Strongly Disagree and 5 = Strongly Agree; ^2^ One sided *t*-test used to test changes after the Food Hero campaign; *p* < 0.05 was considered significant.

**Table 3:** Food Hero Formative Evaluation: Focus Group (FG) and Phone Survey (PS) Demographics.

	Focus Groups 1 (FG-1)	Phone Survey 1 (PS-1)	Focus Groups 2 (FG-2)	Phone Survey 2 (PS-2)
Subjects (*n*)	*n* = 25, 4 FG	*n* = 1244	*n* = 11, 2 FG	*n* = 802
Female (%)	92	86	100	84 (Control) 86 (Intervention)
Mean age (year)	36.7	34.5	42.9	33.2 (Control) 34.7 (Intervention)
Household size (3–4 people) (%)	65	55	36	55 (Control) 54 (Intervention)
Household size (1–2 children) (%)	76	71	64	77 (Control) 71 (Intervention)
Single parent households (%)	52	39	45	45 (Control) 43 (Intervention)

## Abbreviations

The following abbreviations are used in this manuscript:

FV	Fruits and Vegetables
SNAP	Supplemental Nutrition Assistance Program
BRFSS	Behavioral Risk Factor Surveillance System
SNAP-Ed	Supplemental Nutrition Assistance Program—Education
DHS	The Oregon Department of Human Services
PSE	Policy Systems and Environmental change efforts
FG	Focus Group
PS	Phone Survey
SM	Social Marketing
SCT	Social Cognitive Theory
USDA	United States Department of Agriculture
